# *Plasmodium falciparum* gametocyte-infected erythrocytes do not adhere to human primary erythroblasts

**DOI:** 10.1038/s41598-018-36148-y

**Published:** 2018-12-14

**Authors:** Gaëlle Neveu, Florian Dupuy, Meriem Ladli, Daniela Barbieri, Bernina Naissant, Cyrielle Richard, Rafael M. Martins, Jose-Juan Lopez-Rubio, Anna Bachmann, Frédérique Verdier, Catherine Lavazec

**Affiliations:** 1 Inserm U1016, Institut Cochin, Paris, France; 2 Cnrs, UMR8104 Paris, France; 3 Université Paris Descartes, Sorbonne Paris Cité, Paris, France; 4 Laboratoire d’excellence GR-Ex, Paris, France; 5 UMR IRD 224-CNRS 5290-University Montpellier, MIVEGEC, Montpellier, France; 6 Bernhard Nocht Institute for Tropical Medicine, Hamburg, Germany

## Abstract

*Plasmodium falciparum* gametocytes, the sexual stages responsible for malaria parasite transmission, develop in the human bone marrow parenchyma in proximity to the erythroblastic islands. Yet, mechanisms underlying gametocytes interactions with these islands are unknown. Here, we have investigated whether gametocyte-infected erythrocytes (GIE) adhere to erythroid precursors, and whether a putative adhesion may be mediated by a mechanism similar to the adhesion of erythrocytes infected with *P*. *falciparum* asexual stages to uninfected erythrocytes. Cell-cell adhesion assays with human primary erythroblasts or erythroid cell lines revealed that immature GIE do not specifically adhere to erythroid precursors. To determine whether adhesion may be dependent on binding of STEVOR proteins to Glycophorin C on the surface of erythroid cells, we used clonal lines and transgenic parasites that overexpress specific STEVOR proteins known to bind to Glycophorin C in asexual stages. Our results indicate that GIE overexpressing STEVOR do not specifically adhere to erythroblasts, in agreement with our observation that the STEVOR adhesive domain is not exposed at the surface of GIE.

## Introduction

The spread of malaria in population relies on the ability of the *Plasmodium* parasites to be transmitted from infected individuals to mosquito vectors. Understanding the biology of *Plasmodium* gametocytes, the only parasite stages capable of transmission to mosquitoes, is therefore crucial to ablate parasite transmission. For *Plasmodium falciparum*, the causative agent of the most severe form of human malaria, gametocytes maturation lasts about 10 days and is conventionally divided into 5 morphological stages (I–V)^1^. From stage I to IV, immature gametocytes are sequestered away from peripheral circulation, and appear only as mature stages V in the peripheral blood where they are accessible for mosquito bites. Analyses of *ex-vivo* and autopsy specimens from malaria-infected patients revealed that immature gametocytes are predominantly localized in the bone marrow parenchyma, where they are enriched at erythroblastic islands^2–4^. These specialized niches, where the terminal erythroid differentiation occurs, consist of a macrophage surrounded by differentiating erythroblasts^5^.

The unveiling of the hidden sites for gametocytes maturation raised the questions how these parasite stages develop and sequester in the bone marrow micro-environment and how they interact with the erythroblastic islands. Their presence in the bone marrow extra-vascular compartment and the absence of *P*. *falciparum* erythrocyte membrane protein 1 (PfEMP1) expression in gametocytes^6^ suggest that gametocytes sequester via different mechanisms than those of asexual stages, which depend on cytoadhesion of infected erythrocytes to vascular endothelium through the interaction of PfEMP1 with endothelial receptors^7,8^. Moreover, studies of gametocyte-infected erythrocytes (GIE) cytoadhesion failed to show any binding interactions of immature GIE with human endothelial cells from different tissues, including human bone marrow-derived endothelial cell lines^6,9–11^. The proximity of immature GIE with erythroblastic islands suggests that GIE may adhere to erythroid precursors. A binding interaction between these cells may be mediated by a mechanism similar to the adhesion of asexual stages-infected erythrocytes to uninfected erythrocytes. This phenomenon, called “rosetting”, has been linked to disease severity^12^. Although rosetting has been almost exclusively observed in asexual stages, we hypothesize that a “rosetting-like” adhesion may occur between GIE and erythroid precursors. Among the different erythrocyte receptors involved in rosetting of asexual stages, Glycophorin C (GPC) is one of the key host receptors for rosetting with erythrocytes infected with both *P*. *falciparum* and *P*. *vivax*^13,14^. GPC interacts with parasite proteins called STEVOR (SubTElomeric Variable Open Reading frame). These proteins belong to one of the major parasite proteins family and are exposed at the infected erythrocyte surface in late trophozoite stages^14–16^. Interestingly, STEVOR proteins are exported to the infected erythrocyte membrane by immature gametocytes^17,18^, and one study has reported their presence at the surface of live GIE^17^. Moreover, GPC protein expression starts in the early stage of erythropoiesis^19,20^. Therefore, an interaction between the GPC located at the surface of erythroid precursors and STEVOR proteins exposed at the surface of immature GIE would be plausible. Based on this, we hypothesized that the adhesion of STEVORs to GPC might contribute to sequestration of immature gametocytes in the bone marrow parenchyma by promoting adhesion of immature GIE to erythroblasts. Yet, the ability of GIE to cytoadhere to erythroid cells has never been addressed, although one study suggested that GIE from *P*. *falciparum* isolates may be involved in rosette formation^13^. Moreover, STEVORs adhesive properties have never been investigated during sexual stages.

In the present study, we have performed cell-cell adhesion assays to investigate interactions between immature GIE and human primary erythroblasts or erythroid cell lines. To determine the contribution of STEVOR proteins to these interactions, we used clonal lines that express specific *stevor* genes as well as transgenic parasite lines that overexpress or down-regulate members of the *stevor* gene family. Our results show that GIE overexpressing STEVOR do not adhere to erythroblasts, and that STEVOR adhesive domain is not exposed at the surface of GIE.

## Results

### Validation of the cell-cell adhesion protocol

**Figure 1:**
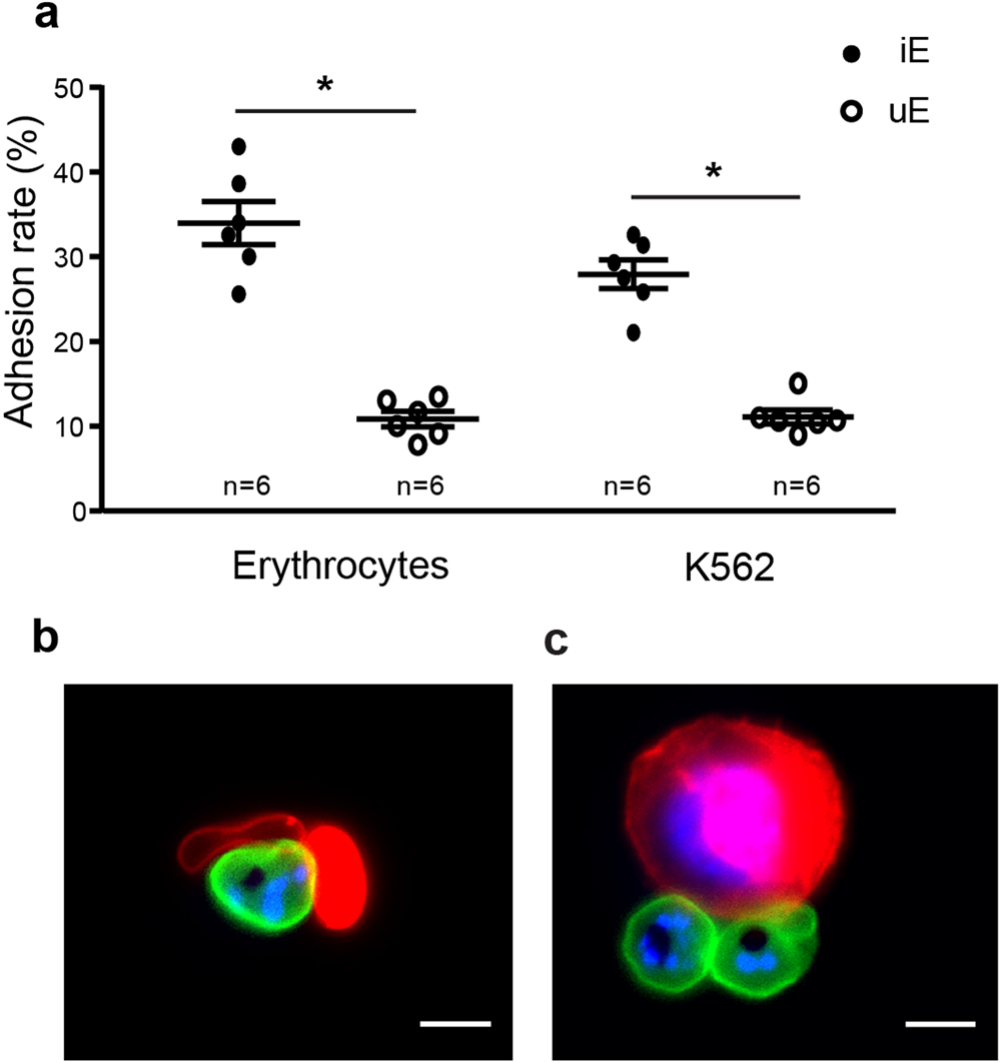
Validation of the cell-cell adhesion protocol. (**a**) Cell-cell adhesion assays of MACS-purified erythrocytes infected with asexual VarO trophozoites (iE) or uninfected erythrocytes (uE) to erythrocytes (p = 0.03) or K562 cells (p = 0.03). Error bars denote the standard error of the mean. n = number of experiments. (**b**,**c**) Live microscopy imaging showing adhesion of VarO-infected erythrocytes stained with PKH67 (green) to uninfected erythrocytes (**b**) or K562 cells (**c**) stained with PKH26 (red). DNA is stained with Hoechst 33342 (blue). Bars represent 5 µm.

The presence of immature gametocytes near the erythroblastic islands in extravascular spaces of bone marrow suggests a direct adhesion of immature GIE to the developing erythroblasts. To test this hypothesis, we set up a cell-cell adhesion protocol with MACS-purified infected erythrocytes and erythroid cells. We first validated our protocol with the VarO parasite line known to adhere to the ABO blood group and commonly used as a model for *in vitro* rosetting^21^. With this adhesion protocol, erythrocytes infected with VarO asexual parasites significantly adhered to non-infected erythrocytes (Fig. 1a,b) and K562 erythroid cells (Fig. 1a,c), indicating that size and rigidity of nucleated erythroid cells are not a hindrance for infected erythrocyte adhesion.

### NF54 GIE fail to adhere to erythroid cells

**Figure 2:**
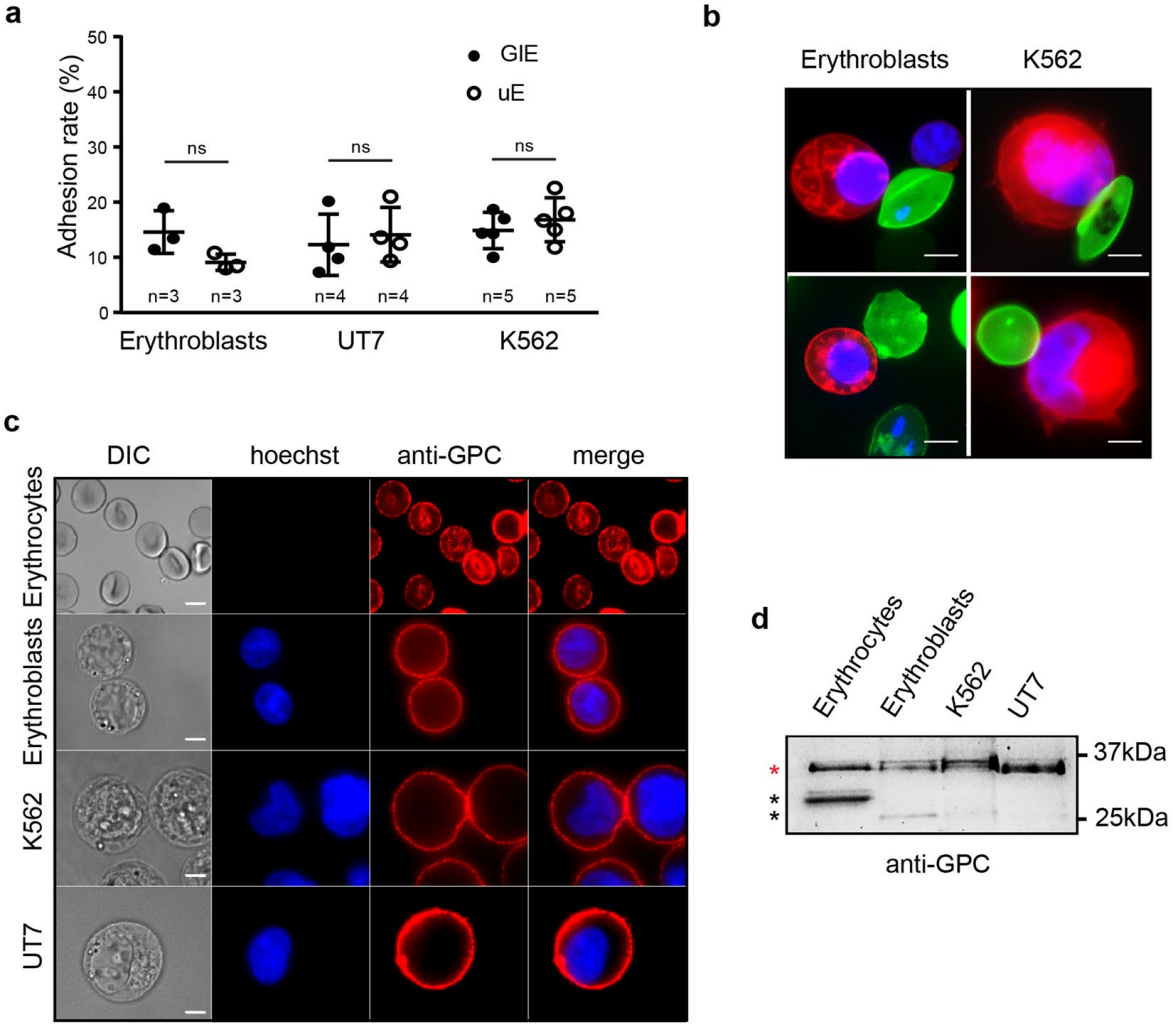
NF54 GIE fail to adhere to erythroid cells. (**a**) Cell-cell adhesion assays of MACS-purified NF54 GIE (GIE) or uninfected erythrocytes (uE) to human primary erythroblasts, UT7 cells and K562 cells. Error bars denote the standard error of the mean. ns, non-significant differences in adhesion rates. n = number of experiments. (**b**) Live microscopy imaging showing non-specific adhesion of MACS-purified NF54 GIE (upper panel) and uninfected erythrocytes (lower panel) stained with PKH67 (green) to human primary erythroblasts or K562 cells stained with PKH26 (red). DNA is stained with Hoechst 33342 (blue). Bars represent 5 µm. (**c**) Immunofluorescence analysis (IFA) of paraformaldehyde-fixed uninfected erythrocytes, erythroblasts, K562 cells and UT7 cells stained with anti-GPC mAb followed by anti-mouse Alexa594-conjugated IgG, showing the presence of GPC at the surface of all erythroid cells. DNA is stained with Hoechst 33342 (blue). Bars represent 5 µm. (**d**) Western blot analysis showing the presence and the size of GPC in erythrocytes, erythroblasts, K562 cells and UT7 cells. Red star indicates the expected size of fully glycosylated GPC (32 kDa). Black stars indicate degradation products. Full-length blot is presented in Supplementary Fig. S1.

We then used our adhesion protocol to address whether immature GIE bind to primary erythroblasts derived from CD34+ progenitor cells from human bone marrow or to the erythroid cell lines K562 and UT7 (Fig. 2a,b). As VarO parasites do not produce gametocytes, we quantified adhesion of immature GIE from the NF54 gametocyte-producing strain. We observed that adhesion rates of MACS-purified immature NF54 GIE to erythroblasts or erythroid cell lines were not significantly different from those of uninfected erythrocytes from the same culture (Fig. 2a), indicating that NF54 GIE do not specifically adhere to erythroid precursors. Since we hypothesized that GIE might adhere to erythroid cells via interaction with GPC, we performed immunostaining of non-permeabilized UT7 cells, K562 cells, erythroblasts and erythrocytes with an anti-GPC antibody to confirm that GPC is properly expressed at the surface of all types of erythroid cells (Fig. 2c). Moreover, immunoblotting analysis detected in all types of cells a major band at 32 kDa that corresponds to the expected size of fully glycosylated GPC^22^, suggesting that glycosylation levels are comparable in erythrocytes and erythroid precursors (Fig. 2d). These results indicate that NF54 immature GIE fail to adhere to erythroid cells expressing GPC. These findings suggest either that GIE and erythroblasts never bind together, or that adhesion is dependent on GIE protein(s) that are not expressed in the NF54 strain.

### Endogenous levels of STEVOR expression do not promote GIE adhesion to erythroid cells

**Figure 3:**
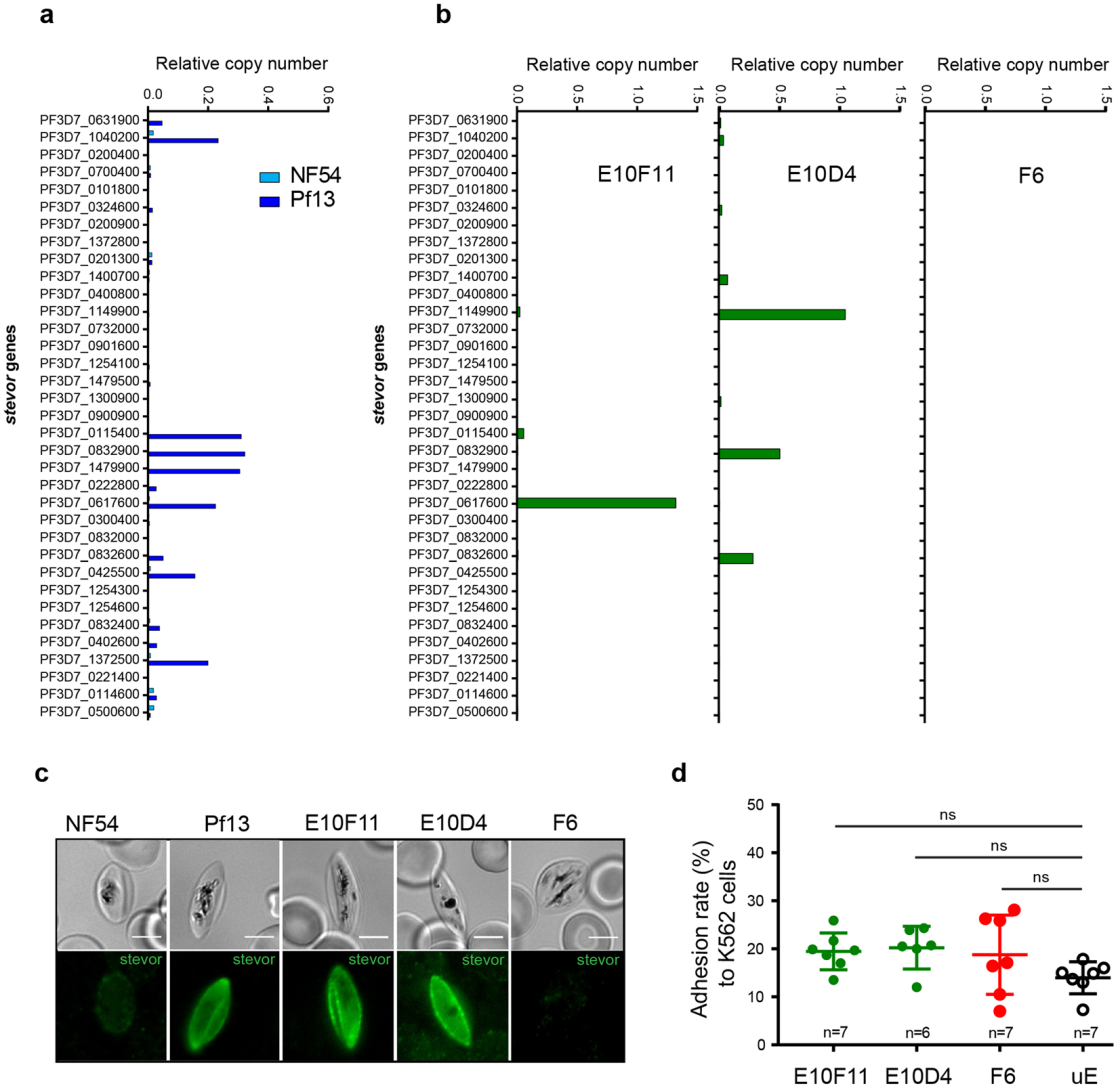
Endogenous levels of STEVOR expression do not promote GIE adhesion to erythroid cells. (**a**,**b**) RT-qPCR analysis of transcriptional levels for the 35 *stevor* genes in trophozoites (28 hours post-infection) of NF54 and 3D7/Pf13 lines (**a**), and of NF54 clones E10F11, E10D4 and F6 (**b**). Transcription levels were normalized to the transcription level of the *arginyltRNA synthetase* reference gene (*PF3D7_1218600*). (**c**) IFA of permeabilized GIE showing the expression of STEVOR protein (green) in NF54, 3D7/Pf13, E10F11, E10D4 and F6 parasite lines. GIE were stained with a rabbit polyclonal antibody directed against STEVOR PF3D7_0631900 protein followed by anti-rabbit Alexa488-conjugated IgG. Pictures were taken under identical exposure conditions. Bars represent 5 µm. (**d**) Cell-cell adhesion assays of MACS-purified GIE from NF54 clones or of uninfected erythrocytes (uE) to K562 cells. Error bars denote the standard error of the mean. ns, non-significant differences in adhesion rates. n = number of experiments.

The absence of specific adhesion between NF54 GIE and erythroid cells may be explained by a too low expression of *stevor* genes in the NF54 polyclonal culture. Indeed, the expression of this multigenic family is clonally variant and undergoes switching^23^, therefore the low level of transcription of *stevor* genes in comparison with the endogenous control gene expression in NF54 trophozoites (Fig. 3a) suggests that only a proportion of the parasite population efficiently transcribes *stevor* genes. Moreover, the STEVOR N-terminal sequence is variable among the 35 members of the STEVOR family, and adhesive properties may not be conserved in the entire family. For instance, only a subset of PfEMP1 variants can mediate rosetting^24^. Since the ability to bind GPC has been addressed for just a few STEVOR members^14^, it is thus possible that the members expressed in NF54 GIE are not involved in adhesion. To determine which STEVOR member(s) are likely involved in rosetting, we analyzed by RT-qPCR the *stevor* genes transcription profile in the 3D7/Pf13 parasite line that has been enriched for a rosetting phenotype^25^ (Supplementary Fig. S2a). In contrast with NF54 parasites, seven *stevor* genes (*PF3D7_1040200*, *PF3D7_0115400*, *PF3D7_0832900*, *PF3D7_1479900*, *PF3D7_0617600*, *PF3D7_0425500*, and *PF3D7_1372500*) were significantly transcribed in trophozoites of this parasite line (Fig. 3a). Among them, four genes (*PF3D7_1040200*, *PF3D7_0115400*, *PF3D7_0832900*, and *PF3D7_0617600*) were selected in a published microarray analysis^14^ as upregulated in two parasite lines enriched for rosetting. To determine the contribution of these STEVOR members to a potential adhesion to erythroid cells, we utilized parasite clones of NF54 that were previously characterized for *stevor* expression^23^, instead of the 3D7/Pf13 line that poorly generates gametocytes (Supplementary Fig. S2b). In particular, we picked two clones which expressed one *stevor* detected in both our RT-qPCR analysis of the 3D7/Pf13 parasite line and the published microarray analysis, and one clone which did not express any *stevor* (Fig. 3b). Immunostaining of immature GIE from these clones with specific anti-STEVOR antibodies confirmed that STEVOR proteins are expressed in E10F11 and E10D4 gametocytes whereas no expression was detected in the F6 clone (Fig. 3c). However, adhesion assays did not show any difference in GIE adhesion to K562 cells between these three clones and uninfected erythrocytes (Fig. 3d). These results suggest that the STEVOR members expressed in E10F11 and E10D4 clones (PF3D7_0617600 and PF3D7_0832900) may not be the ones responsible for the rosetting phenotype in the 3D7/Pf13 line, since we cannot exclude that these proteins may not have adhesive properties. Also, endogenous expression of the selected *stevor* genes may not be sufficient to promote adhesion to erythroid cells. In support of this hypothesis, previous studies have reported a reduction of STEVOR expression in laboratory-adapted parasite lines compared to parasites directly isolated from patients where STEVOR may play a crucial role in host-parasite interaction^26,27^.

### Overexpression of STEVOR does not promote GIE adhesion to erythroid cells

**Figure 4:**
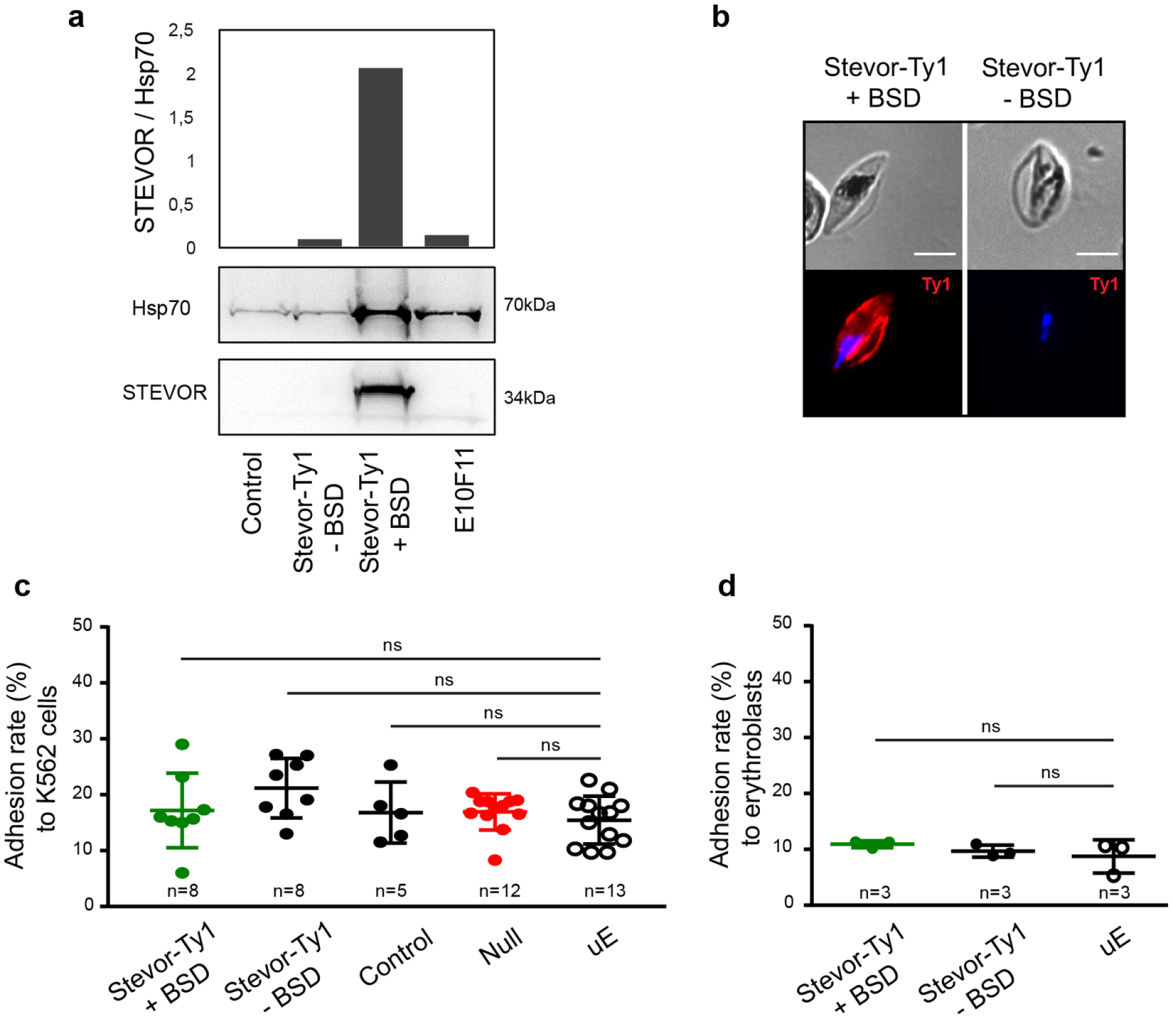
Overexpression of STEVOR does not promote GIE adhesion to erythroid cells. (**a**) Western blot analysis of STEVOR expression in stage III GIEs from the transgenic Stevor-Ty1 line cultivated with and without BSD, the transfection control line (Control) and the NF54 clone E10F11. Immunoblots were probed with a rabbit polyclonal antibody directed against STEVOR PF3D7_0300400 protein and with a mAb directed against PfHsp70. Quantification of STEVOR level relative to the loading control PfHsp70 was performed by densitometry (ImageJ software). Decrease of STEVOR expression in Stevor-Ty1 GIE cultivated without BSD indicates a loss of episomal expression of the epitope-tagged STEVOR protein. Full-length blots are presented in Supplementary Fig. S3. (**b**) IFA of methanol-fixed blood smears showing the expression and erythrocyte membrane localization of PF3D7_1040200 -Ty1 STEVOR protein (red) in Stevor-Ty1 GIE cultivated with and without BSD. GIE were stained with anti-Ty1 mAb followed by anti-mouse Alexa594-conjugated IgG, and parasite nuclei were counterstained with Hoechst 33342. Pictures were taken under identical exposure conditions. Absence of Ty1 expression in Stevor-Ty1 GIE cultivated without BSD indicates a loss of episomal expression of the epitope-tagged STEVOR protein. Bars represent 5 µm. (**c**,**d**) Cell-cell adhesion assay of MACS-purified transgenic Stevor-Ty1 GIE cultivated with and without BSD, of transfection control GIE (Control), of a transgenic GIE downregulating STEVOR expression (Null) or of uninfected erythrocytes (uE) to K562 cells (**c**) or to human primary erythroblasts (**d**). Error bars denote the standard error of the mean. ns, non-significant differences in adhesion rates. n = number of experiments.

To rule out the hypothesis that endogenous expression of *stevor* genes is not sufficient to promote adhesion, we generated a transgenic parasite line (Stevor-Ty1) that episomally overexpresses a Ty1-tagged copy of *stevor* driven by the constitutive promotor of *calmodulin*. To exclude the possibility that the STEVOR members expressed in E10F11 and E10D4 clones are not functional, we opted for the overexpression of the *PF3D7_1040200 stevor* gene. This gene has been selected in our RT-qPCR analysis of the 3D7/Pf13 parasite line, and the PF3D7_1040200 protein has also been previously shown to mediate adhesion of asexual parasite-infected erythrocytes to GPC^14^. As a control, we promoted the shedding of the episome-encoded *PF3D7_1040200* by cultivating the Stevor-Ty1 line for several generations without blasticidin selection (-BSD) required to retain the episome. The transgenic Control parasite line^28^, transfected with an empty plasmid, and the Null parasite line^28^ that down-regulates the entire *stevor* gene family, were also used as negative controls. Immunoblotting and immunostaining of immature GIEs with antibodies against STEVOR and Ty1 tag indicated that STEVOR proteins were twenty-fold more expressed in the Stevor-Ty1 transgenic line than in the E10F11 clone (Fig. 4a) and that PF3D7_1040200-Ty1 was properly exported to the infected erythrocyte membrane (Fig. 4b). However, adhesion assays did not show any significant adhesion of immature GIE from this overexpressing parasite line to K562 cells (Fig. 4c) or to primary erythroblasts (Fig. 4d) compared to the control lines and uninfected erythrocytes. Similar results were obtained with another transgenic line overexpressing the PF3D7_0631900 protein^28^ (Supplementary Fig. S4). These results indicate that overexpression of a STEVOR protein involved in adhesion to GPC in asexual stages does not promote adhesion of gametocyte stages to erythroid cells.

### STEVOR adhesive domain is not exposed at the GIE surface

**Figure 5:**
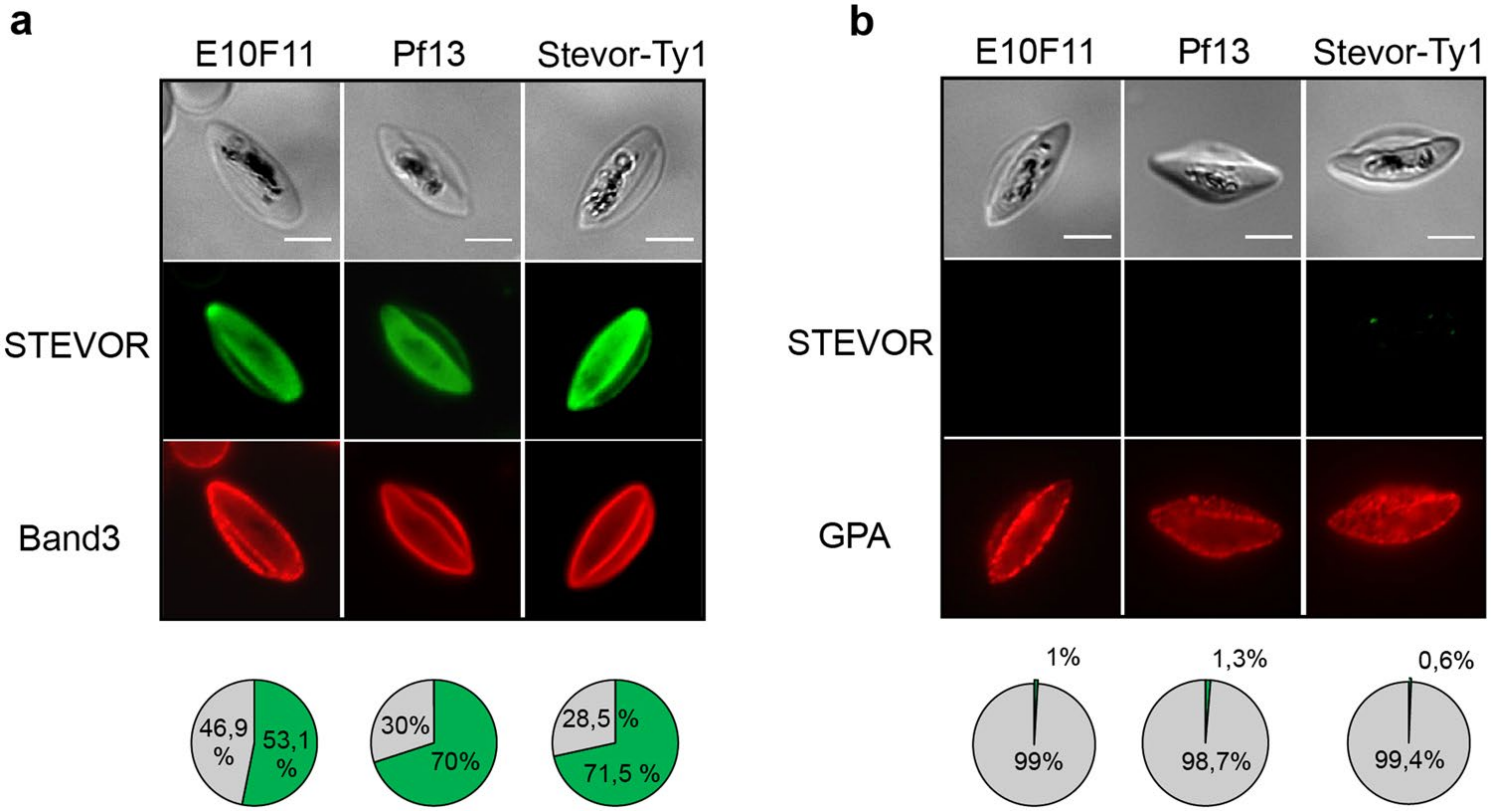
STEVOR proteins are not exposed at the GIE surface IFA of permeabilized (**a**) and non-permeabilized (**b**) immature GIE from the E10F11, 3D7/Pf13 and Stevor-Ty1 (+BSD) parasite lines. GIE were stained with a rabbit polyclonal antibody directed against the N-terminal domain of STEVOR PF3D7_0631900 (green) and with a mouse antibody directed against the cytoplasmic domain of Band 3 ((**a**), red) or with a mouse anti-Glycophorin A (GPA) (**b**, red). Pie charts show proportions of GIE positive (green) or negative (grey) for STEVOR staining. 300 cells per condition were examined. Band 3 and GPA staining control permeabilization and presence of the erythrocyte membrane, respectively. Pictures were taken under identical exposure conditions. Bars represent 5 µm.

Failure of GIE to adhere to erythroid cells may be due to a different conformation of STEVOR proteins at the GIE membrane compared to asexual stages. To address whether the adhesive domain of STEVOR proteins is properly exposed at the surface of erythrocytes infected with gametocytes, we performed IFA on permeabilized and non-permeabilized immature GIE with antibodies directed against the N-terminus domain of STEVOR. This domain includes the variable domain, the first hydrophobic stretch and the semi-conserved region that promotes binding to GPC in asexual stages^14^. Unexpectedly, we observed that antibodies detected the STEVOR N-terminus domain in more than 53.1% of permeabilized cells (Fig. 5a) but in less than 1% of non-permeabilized cells (Fig. 5b) from the E10F11 clone, suggesting that the STEVOR adhesive domain may not be exposed at the surface of GIE. Furthermore, the few non-permeabilized cells positive for STEVOR were either recognized by antibodies directed against a cytoplasmic domain of Band 3, indicating the leakiness of the erythrocyte membrane, or were not recognized by anti-Glycophorin A antibodies, suggesting a loss of the erythrocyte membrane (Supplementary Fig. S5). Similar staining patterns were observed in GIE from the Stevor-Ty1 transgenic line and the 3D7/Pf13 parasite line, indicating that STEVOR exposure at the erythrocyte surface is not dependent on STEVOR expression level or parasite enrichment for rosetting (Fig. 5). Therefore, the missing of STEVOR adhesive domain at the erythrocyte surface likely accounts for the lack of GIE adhesion to erythroid cells.

## Discussion

Deciphering the interactions between *P*. *falciparum* gametocytes and erythroblastic islands may provide insights into the parasite sequestration mechanisms in the bone marrow parenchyma. In the present study, we investigated whether immature GIE adhere to erythroid precursors. Our results indicated that GIE from the NF54 strain fail to adhere to erythroblasts, suggesting either that these cells never bind together, or that adhesion is dependent on protein(s) which are not expressed in the NF54 strain, as for proteins encoded by multigenic families. In support to this latter hypothesis, other adhesion phenotypes in *P*. *falciparum* are often mediated by antigenically variant proteins, such as PfEMP1 proteins that promote adhesion to endothelial cells or RIFIN (Repetitive Interspersed Family) and STEVOR proteins that are involved in asexual stages rosetting^29^. Therefore, we have investigated whether GIE may adhere to erythroid precursors via a mechanism similar to the rosetting mediated by STEVOR and GPC in asexual stages. Our results established that STEVOR proteins are not exposed on the GIE surface, thus making them unlikely candidates for binding to erythroid cells.

These results contrast with data reported for asexual stages where 10 to 13% of erythrocytes infected with schizonts are recognized with antibodies directed against the N-terminus domain of STEVOR^14–16,30^. In a previous study, STEVOR proteins have also been observed at the surface of live GIE^17^, however antibodies used as controls for erythrocyte membrane integrity in this study were directed against the parasite (anti-Pfg27 antibodies) and may not be able to detect a low leakiness of the erythrocyte membrane as we observe here with anti-Band 3 antibodies. Despite the absence of surface exposure, we observed STEVOR proteins in association with the GIE membrane (Figs 3c, 4b and 5a), in agreement with previous studies^17,18,28^. These observations are consistent with a model of a two-transmembrane topology at the GIE membrane with a surface-exposed short hypervariable region and C-terminal and N-terminal regions facing the erythrocyte cytoplasm^31,32^. Further studies are needed to validate this predicted topology and confirm that it differs from the topology with only one transmembrane domain observed in asexual stages. It is tempting to speculate that the potential different topologies in asexual and sexual stages may reflect different functions of the protein and may reveal a strategy that the parasite has evolved to differently modify its host cell to adapt to different microenvironments. For instance, it has been proposed that the STEVOR-mediated stiffness of immature GIE may contribute to their sequestration by mechanical retention in the bone marrow parenchyma^17,33^. Since the increased erythrocyte stiffness depends on the interaction between the C-terminus domain of STEVOR and the erythrocyte cytoskeletal ankyrin complex^28^, our results suggest that the STEVOR N-terminus domain may also interact with cytoskeletal proteins and contribute to the rigidification of the erythrocyte membrane. This interaction may occur in immature GIE but not in asexual stages where sequestration is mediated by infected cell adhesion to endothelium rather than mechanical retention. On the other hand, surface-exposed STEVOR proteins preferentially localize in proximity to knobs in asexual stages, and this localization has been proposed to confer an advantage to the parasite by reinforcing the cytoadherence of infected erythrocytes^16^. It is plausible that surface-exposure of the STEVOR adhesive domain is dependent on knobs formation, therefore the absence of knobs in gametocytes stages^6^ may result in a the two-transmembrane topology leading to absence of STEVOR at the surface. Nevertheless, we cannot exclude that STEVOR exposure and/or STEVOR-mediated adhesion requires an additional co-factor expressed *in vivo* but not *in vitro*, or not expressed in our parasite lines.

Other ligand(s) and receptor(s) may also promote adhesion between GIE and erythroblastic islands. For instance, the parasite proteins GEXP07 and GEXP10 exhibit adhesive properties and are exposed at the surface of erythrocytes infected with early gametocytes^34^. However this hypothesis is not supported by our results showing the absence of adhesion between several types of erythroid cells and NF54 GIE that constitutively express GEXP proteins. In contrast, other clonally variant adhesins expressed by multigenic families, such as RIFIN^35^, may be involved in GIE adhesion to erythroid cells since they mediate rosetting in asexual stages via binding to the blood group A antigen and to the Glycophorin A^36^. Their presence in immature gametocytes^37^ makes them interesting ligand candidates that need to be investigated in further studies.

It remains to be clarified how gametocytes sequester in the bone marrow parenchyma. The proposed contribution of infected erythrocyte stiffness to this phenotype is supported by recent data showing that sildenafil citrate, a phosphodiesterase inhibitor known to keep GIE in a rigid state^38^, increases the enrichment of *Plasmodium berghei* gametocytes in the bone marrow of infected mice^39^. It is also possible that immature GIE adhere to non-erythroid bone marrow cells. Interestingly, recent work revealed that erythrocytes infected with immature gametocytes or with asexual stages adhere to human bone marrow mesenchymal cells via trypsin-sensitive parasite ligands exposed on the erythrocyte surface^40^. Although the ligand(s) and receptor(s) promoting this adhesion are not identified yet, this mechanism may contribute to immature GIE sequestration in the parenchyma. Furthermore, we cannot exclude that the association of immature gametocytes with erythroblastic islands reflects the infection of erythroblasts by gametocytes. Indeed, several experiments using *ex vivo* culture of human primary erythroblasts showed that the development of gametocytes could take place in hematopoietic cells as early as the orthochromatic stages^4^, suggesting that gametocytes may preferentially develop in erythroblasts or reticulocytes in the human marrow.

In conclusion, this study provides insights into the interplay between *P*. *falciparum* gametocytes and human bone marrow cells. Also, our results highlight that STEVOR proteins may have different role and topology in asexual and sexual stages. These observations raise some questions about the function of other variant antigens in gametocytes.

## Methods

### Parasite culture

The VarO and the 3D7/Pf13 parasite lines were described elsewhere^21,25^. The *P*. *falciparum* isolate NF54^41^ was used to generate clonal and transgenic lines (see Supplemental Table 1 for description of parasite lines). Parasites were cultivated *in vitro* as described^42^ using RPMI 1640 medium (Gibco) supplemented with 10% heat-inactivated human serum, hypoxanthin 10 mM, gentamicin (20 µg/ml) and human erythrocytes at 5% hematocrit. Infected erythrocytes were cultivated at 37 °C, 5% CO2, 2% O2. To obtain synchronous asexual stages, parasites were synchronized by the isolation of trophozoites by magnetic isolation using a MACS depletion column (Miltenyi Biotec) in conjunction with a magnetic separator, and placed back into culture. After the invasion of merozoites, ring-stage parasites were selected by magnetic depletion of trophozoites to obtain a tighter window of synchronization. Synchronous production of gametocytes was achieved as described^43^. Briefly, the culture medium was supplemented with 50 mM N-acetyl-glucosamine from day 0 onwards, and a medium replacement was continued for 5 to 6 days to eliminate the asexual stages.

### DNA construct and transfection

To overexpress the PF3D7_1040200-tagged protein having two adjacent Ty1 at the carboxy-terminal domain, the plasmid pLN-*stevorPF3D7_1040200*-Ty1 was constructed by amplifying the *PF3D7_1040200* gene from NF54 genomic DNA using these primers: Fw, 5′-AAGGCCGGCCCTCGAGATGAAGATGTATTACCTTAAAATGATATTGTTTAAC and Rv, 5′- AAGGCGCGCCACTTACATAAATGTTTCTTGCATTGATGTTTCAATGAATTTTTTC. The PCR product was restriction digested with FseI and AscI and inserted into the expression vector pLN-Ty1C^44^. This expression vector contains an expression cassette that is driven by the *P*. *falciparum* 5′ *calmodulin* promoter and the 3′ *hsp86* terminator, to ensure a high expression level of *stevor* gene, and *blasticidin* (bsd) expression cassette for selection of transformed parasites. Sequence of the plasmid was confirmed by sequencing: Fw, 5′-TGTATATTGGGGTGATGATAAAATG and Rv, 5′-AATATGTATATTTTAAACTAGAAAAGG. Parasites were transfected into the NF54-derived B10 clone^17^ as previously described, by electroporating ring-stage parasites^45^. Briefly, 0.2 cm electroporation cuvettes were loaded with 0.100 mL of ring-stage infected erythrocytes and 60 μg of plasmid DNA in cytomix solution. Positive selection was achieved using 2.5 μg/mL blasticidin (Euromedex). Episomal plasmid rescue experiment was performed by transforming *E*. *coli* competent cells with 100 ng of purified *P*. *falciparum* genomic DNA. To remove episomal overexpression, parasites were cultivated without blasticidin for 14 generations.

### Erythroid Cell Culture

The K562 cell line was cultured in RPMI 1640 (Gibco) supplemented with 2 mM L-glutamine, 200 U/mL penicillin, 200 mg/mL streptomycin and 10% heat-inactivated fetal bovine serum^46^. The UT7 cell line was cultured in minimal essential medium (MEM) (Gibco) supplemented with 2 U/mL erythropoietin (Epo), 2 mM L-glutamine, 200 U/mL penicillin, 200 mg/mL streptomycin and 10% heat-inactivated fetal bovine serum^47^. The human primary erythroblasts culture method was adapted from Freyssinier *et al*.^48^. CD34+ progenitor cells were obtained from human donors who gave informed consent in accordance with the Declaration of Helsinki Principles. The study has been approved by the French ministry of higher education and research review board (DC-2012–1645). CD34+ cells were collected from G-CSF mobilized peripheral blood after cytapheresis and purified by an immunomagnetic procedure (MACS CD34 isolation Kit; Miltenyi Biotech). CD34+ cells were cultured in 5% CO2 at 37 °C in Iscove DMEM (IMDM) containing 15% BIT 9500 (StemCell Technologies, Vancouver, Canada), 100 ng/mL Stem Cell Factor (SCF, Milteny Biotech), 10 ng/mL IL6 and 10 ng/mL IL3. After 7 days of culture, a purified population of human erythroid progenitors was obtained by positive selection on CD36 immunomagnetic beads. CD36+ cells were cultured with 2 U/mL Epo, 100 ng/mL SCF and 10 ng/mL IL3 to allow the erythroid differentiation.

### Adhesion assay

Infected-erythrocytes were purified by magnetic isolation using a MACS depletion column as described above. Purified infected-erythrocytes were stained with PKH67 (Sigma) according to manufacturer’s instructions and Hoechst 33342 (Life Technologies) (1/20000). Erythroid cells were stained with PKH26 and Hoechst 33342. 20% of purified infected-erythrocytes were added to 80% of erythroid cells. Cells were allowed to adhere for 2 hours in 100% human serum at 37 °C, 5% CO_2_, 2% O_2_. After incubation, at least 300 cells per condition were counted at the X100 objective of a Leica DMi8 microscope. Statistical significances of adhesion rates were determined with the Wilcoxon test, and raw p-values were adjusted for multiple testing using the Benjamini-Hochberg procedure^49^.

### Antibodies

The 3D7 genomic DNA sequence coding for the N-terminal part including the semi-conserved region, the first hydrophobic stretch and the variable domain of STEVOR PF3D7_0631900 was amplified using the following primers: Fw: 5′-GGGAAGCTTACCCAAATCCATAATCCAC-3′; Rv: 5′-GGGGGATCCTTAAGGGGGAAATGCACCTATAGC-3′). Recombinant protein was produced in *E*. *coli* and antisera directed against the protein was produced in rabbits (Pineda Antikörper-Service).

### Immunofluorescence assays (IFA)

To detect the GPC at the surface of nucleated erythroid cells and erythrocytes were fixed during 10 minutes with a solution of 1X Phosphate-Buffered-Saline (PBS)/4% Paraformaldehyde (PFA) or 1% PFA, respectively. After 2 hours of pre-incubation in 1X PBS/2% Bovine Serum Albumin (BSA), slides were incubated overnight with a mouse monoclonal antibody directed against Glycophorin C/D (Santa Cruz) (1/200).

To assess STEVOR protein expression in the different parasite lines, GIE were fixed with a solution of 1X PBS/1% PFA/0.25% glutaraldehyde for 10 minutes and permeabilized with a solution of 1X PBS/0.1% Triton (Sigma) for 10 minutes or GIE were air-dried on glass blood smears and methanol-fixed at −20 °C for 10 minutes. After 2 hours of pre-incubation in 1X PBS/2% BSA, slides were incubated overnight with rabbit antibodies raised against STEVOR PF3D7_0300400^28^ (1/200), with a mouse antibody raised against the intracellular domain of Band 3 protein (Santa Cruz) (1/10000) or with a mouse mAb directed against Ty1 (Diagenode) (1/50000).

For live surface IFA, GIE were incubated 1 hour at 37 °C with antibodies directed against STEVOR PF3D7_0300400 (1/200), the N-terminal domain of Glycophorin A/B (Santa Cruz) (1/500) and the intracellular domain of Band 3 protein (Santa Cruz) (1/10000).

All incubations with primary antibodies are followed by incubation with Alexa Fluor 488-conjugated goat anti-rabbit (1/2000) and Alexa Fluor 594-conjugated goat anti-mouse antibody (1/2000) and Hoechst 33342 (Life technologies) (1/20000) for 1 hour at room temperature. Samples were observed at X100 magnification using a Leica DMi8 microscope.

### Western-blotting analysis

For the detection of the GPC in erythroid cells, proteins were extracted from whole cells with a RIPA buffer (Sigma) in the presence of protease inhibitor cocktail (Roche). Protein concentration was determined using a Pierce BSA assay kit (Thermo Scientific). 30 to 60 µg of proteins (depending on cell types) were denatured in protein loading buffer (Biorad) during 10 min at 95 °C and were separated by 4–12% SDS-PAGE, transferred to PVDF membrane and blocked 2 hours in 5% non-fat dry milk. Immunoblots were probed overnight with primary mouse GPC antibody (Santa Cruz) (1/500) followed by horseradish peroxidase-conjugated anti-mouse or IgG secondary antibodies (Promega) (1/10000) for 1 hour. Detection step was performed using the Pierce™ ECL Western Blotting Substrate (Thermo Scientific) following the manufacturer’s instructions.

To characterize transgenic parasite lines, stage III GIE were purified by magnetic isolation, pelleted by centrifugation at 1,800 rpm and kept at −80 °C until used. After protein separation and transfer to PVDF membrane, immunoblots were probed overnight with rabbit antibodies against STEVOR PF3D7_0300400^28^ (1/2000) and mouse anti-PfHsp70^28^ (1/5000) followed by horseradish peroxidase-conjugated anti-mouse and anti-rabbit IgG secondary antibodies (Promega) (1/10000) for 1 hour. Detection step was performed using the Pierce™ ECL Western Blotting Substrate (Thermo Scientific) following the manufacturer’s instructions. The levels of STEVOR were quantified by densitometry using the ImageJ software by determining the ratio of STEVOR level relative to the loading control PfHsp70.

### RT-qPCR

RNA was extracted from mid-trophozoite stages at 28 hours post-invasion (hpi). RNA was prepared with Trizol (Invitrogen) as recommended by the manufacturer and treated with DNAse I (Roche). RNA was then reverse-transcribed using Superscript III that was primed with random hexamer primers (Invitrogen). The absence of genomic DNA contamination was confirmed by PCR amplification with *arginyl-tRNA synthetase* primers (*PF3D7_1218600)* on RNA samples that lacked reverse-transcriptase. Real-time PCR was performed using a Light Cycler 480 (Roche). Reactions were prepared in 10 μL volumes using SYBR Green PCR master mix (Roche) and 1 µM primers. Relative quantification of cDNA was performed using the standard curve method to determine the efficiency of the target and reference amplification and to quantify cDNA in each sample. The relative copy number for each gene was determined as the ratio of the relative amount of target gene cDNA/the relative amount of housekeeping control gene cDNA *arginyl-tRNA synthetase* (*PF3D7_1218600*). The threshold detection level (TDL) was defined as 3 CT below the CT value that was obtained for the water control or RNA samples treated without reverse-transcriptase. Gene-specific primers for *stevor* genes and housekeeping genes were published elsewhere^23^.

## Electronic supplementary material

Supplemental information
