# Supplementary material for: Plasmodium falciparum gametocyte-infected erythrocytes do not adhere to human primary erythroblasts
Source: Sci Rep. 2018 Dec 14;8:17886. doi: 10.1038/s41598-018-36148-y (PMC6294825; doi:10.1038/s41598-018-36148-y)
Supplement: Supplementary file 1 — Supplemental information [file 41598_2018_36148_MOESM1_ESM.pdf]

***Plasmodium falciparum* gametocyte-infected erythrocytes do not adhere to human primary erythroblasts**

Gaëlle Neveu, Florian Dupuy, Meriem Ladli, Daniela Barbieri, Bernina Naissant, Cyrielle Richard, Rafael M. Martins, José-Juan Lopez-Rubio, Anna Bachmann, Frédérique Verdier, Catherine Lavazec.

Supplemental Figures and legends

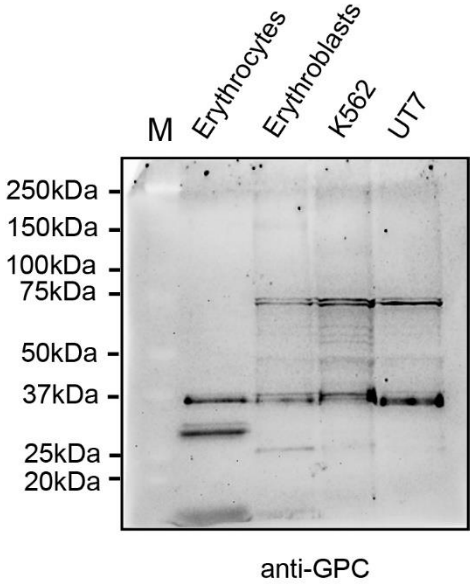

Supplemental Figure S1. Western blot analysis showing the presence and the size of GPC in erythrocytes, erythroblasts, K562 cells and UT7 cells. The full-length blot is presented. Detection step was performed using the Pierce™ ECL Western Blotting Substrate (Thermo Scientific) following the manufacturer's instructions. Image was taken with Fusion FX (Vilber Lourmat). M: molecular weight.

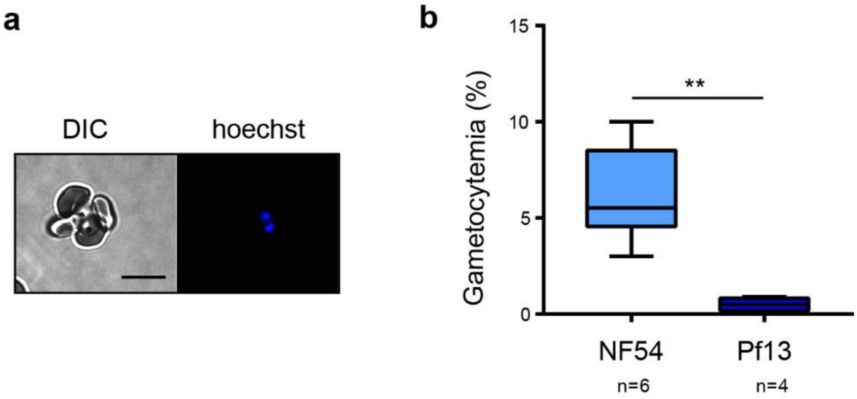

Supplemental Figure S2. 3D7/Pf13 asexual parasites form rosettes and poorly generate gametocytes.

(a) Live microscopy imaging showing rosetting of trophozoites from 3D7/Pf13 parasite line in differential interference contrast (DIC) (left) and stained with Hoechst (right). Bar represents 5µm. (b) Gametocytemia of NF54 and 3D7/Pf13 parasite lines at immature stages (stages II and III) were determined on Giemsa-stained blood smears ($p = 0.0020$, unpaired t-test). Error bar denotes the standard error of the mean. n = number of experiments.

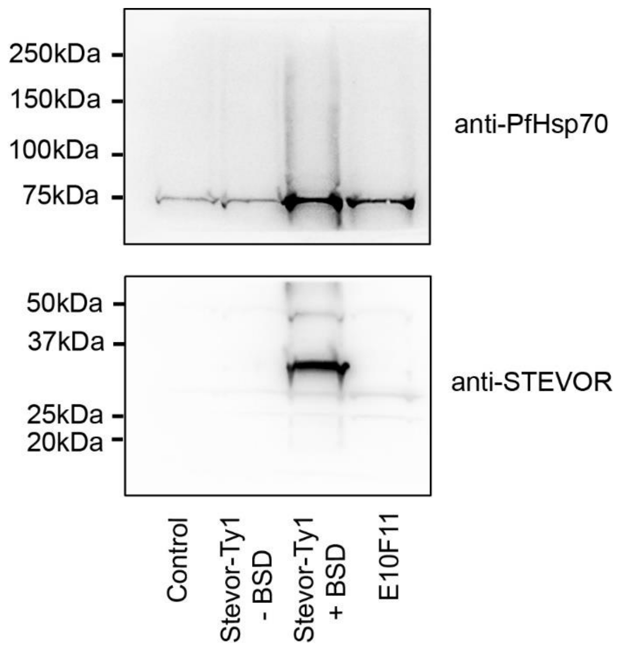

Supplemental Figure S3. Western blot analysis of STEVOR expression in stage III GIEs. 5.10^6 stage III GIE from the transgenic Stevor-Ty1 line cultivated with and without BSD, the transfection control line (Control) and the NF54 clone E10F11 were loaded on one gel. After protein separation and transfer to PVDF membrane, the immunoblot was cut in two pieces. The upper one was probed with a mAb directed against PfHsp70 (upper panel) and the lower one with a rabbit polyclonal antibody directed against STEVOR PF3D7_0300400 protein (lower panel). Full-length blots are presented. Detection step was performed using the Pierce™ ECL Western Blotting Substrate (Thermo Scientific) following the manufacturer's instructions. Images were taken with Fusion FX (Vilber Lourmat).

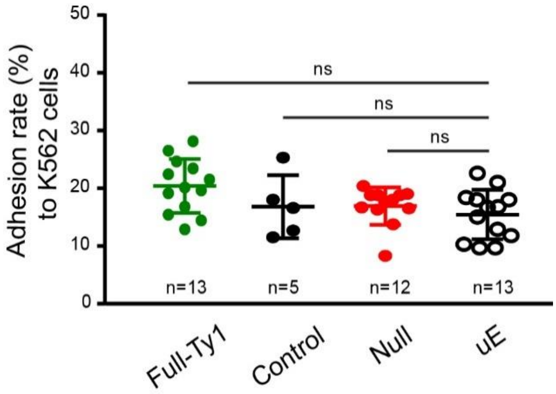

Supplemental Figure S4. Overexpression of STEVOR PF3D7_0631900 does not promote GIE adhesion to K562 cells

Cell-cell adhesion assay of MACS-purified GIE from the transgenic line Full-Ty1, the transfection control line (Control), the transgenic line downregulating STEVOR expression (Null) or of uninfected erythrocytes (uE) to K562 cells. The Full-Ty1 line, characterized in Naissant *et al.*[27], episomally overexpresses a Ty1-tagged copy of the PF3D7_0631900protein exported at the GIE membrane. Error bars denote the standard error of the mean. ns, non-significant differences in adhesion rates. n = number of experiments.

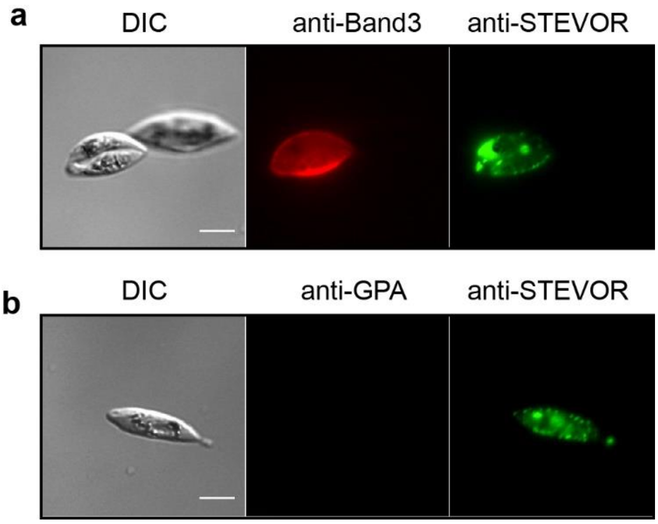

Supplemental Figure S5. Immunofluorescence analysis of STEVOR expression in non-permeabilized GIE

IFA of non-permeabilized immature GIE showing the detection of STEVOR protein (green) in 1 % cells from the E10F11 clone. GIE were stained with a rabbit polyclonal antibody directed against STEVOR PF3D7_0631900 (green) and with a mouse anti-Band 3 (a, red) or a mouse anti-Glycophorin A (GPA) (b, red) antibody. Band 3 positive signal indicates leakiness of the erythrocyte membrane (a), and absence of GPA signal indicates loss of the erythrocyte membrane (b). Pictures were taken under identical exposure conditions. Bars represent 5 µm.

Parasite line	Derived from	Generated by	Clonal	*stevor* expression	Gametocyte production	Cultured under drug	Described in
**NF54**	Field isolate	-	no	low	yes	no	ref [40]
**VarO**	Palo Alto	limiting dilution	yes	not determined	no	no	ref [21]
**3D7/Pf13**	3D7	positive selection for the Pf13 variant	no	ON :7 genes (see Fig. 3)	poor	no	ref [25]
**E10F11**	NF54	limiting dilution	yes	ON : *PF3D7_0617600*	yes	no	ref [23]
**E10D4**	NF54	limiting dilution	yes	ON : *PF3D7_0832900* *PF3D7_0425500*	yes	no	ref [23]
**F6**	NF54	limiting dilution	yes	OFF	yes	no	ref [23]
**Stevor-Ty1 + BSD**	NF54	transfection	no	ON, overexpression : *PF3D7_1040200*	yes	blasticidin	new line
**Stevor-Ty1 - BSD**	NF54	episome shedding	no	low	yes	no	new line
**Control**	NF54	transfection	no	low	yes	blasticidin	ref [28]
**Null**	NF54	transfection	no	OFF, downregulation	yes	blasticidin	ref [28]
**Full-Ty1**	NF54	transfection	no	ON, overexpression : *PF3D7_0631900*	yes	blasticidin	ref [28]

Supplemental Table 1. Description of different parasite lines
